# Using a Standardized Protocol to Assess Female Codling Moth, *Cydia pomonella* (L.), Mating Status Under Mating Disruption Technologies

**DOI:** 10.3390/insects17010099

**Published:** 2026-01-15

**Authors:** Alan Lee Knight, Michele Preti, Esteban Basoalto

**Affiliations:** 1 Instar Biologicals, Yakima, WA 98908, USA; 2 Independent Integrated Pest Management Consultant and Researcher, 48018 Faenza, Italy; 3 Facultad de Ciencias Agrarias y Alimentarias, Universidad Austral de Chile, Valdivia 5090000, Chile

**Keywords:** pear ester, (*E*,*Z*)-2,4-ethyl decadienoate, kairomones, female monitoring, unmated females, sterile moths

## Abstract

Implementing mating disruption (MD) programs to manage codling moth (CM), *Cydia pomonella* (L.), should be based on knowledge of how effectively each program disrupts female mating. A recent survey of 142 pome fruit orchards under MD in Washington State and Oregon found that, on average, about half of the CM females caught in traps baited with a kairomone-based lure were mated. However, significantly lower proportions of mated females were sampled when the intensity of the MD program was increased. A standardized protocol that could reduce the large inter-orchard variability was developed, involving weekly releases of sterilized CM adults. Eleven trials were conducted in 2023 and 2024 across 82 orchards treated with 20 MD programs. The intensive MD programs were significantly more effective in reducing mating of both wild and sterile CM females. Three advantages of using sterile moths to assess CM MD were identified: (*i*) it minimized the impact of wild immigrant females or individuals previously exposed to sublethal spray residues; (*ii*) it allowed greater numbers of females to be dissected, thus increasing the precision of the mean value; and (*iii*) it and allowed the collection of sufficient sampling data (>5 CM females per site) from 30% more orchards than relying on wild moth catch.

## Simple Summary

Codling moth (CM) is a worldwide pest of apples and pears, feeding directly on the fruit. Growers have widely adopted the use of mating disruption (MD) with various formulations to help manage CM, but surprisingly have not carefully assessed the mating status of wild females. A new dual-sex non-pheromone lure was recently used in 2021–2022 to ascertain the level of female mating across 142 pome fruit orchards. The proportion of females mated varied widely among MD-treated orchards, with the median proportion of unmated females being <0.50, only ~0.20 higher than in untreated orchards. Following this initial study, 20 different MD programs were assessed in 82 orchards during 2023–2024, including programs using more than one MD-based tactic. Mark–recapture of sterilized moths was used to minimize orchard variability due to CM immigration. The most effective programs had >75% and >90% unmated wild and sterilized females, respectively. These more effective MD programs may be useful for organic orchards of premium cultivars with concerns for effective CM management.

## 1. Introduction

Monitoring the mating status of female codling moth (CM), *Cydia pomonella* L. (Lepidoptera: Tortricidae), allows a direct assessment of mating disruption (MD) programs. A recent review has drawn attention to efforts to quantify the level of female mating in field studies with four important tortricid moth pests of tree fruits and grapes [1]. Surprisingly, the estimated level of mating was divergent based on whether the sampling method was a preselected canopy position of a laboratory-reared virgin female tethered or placed inside a sticky trap versus the active catch of wild mated and unmated females flying into traps, such as clear interception traps or traps baited with a dual-sex lure (pear ester, (*E*,*Z*)-2,4-ethyl decadienoate) for CM. The passive sampling methods suggested that CM MD can be effective in preventing mating, with results > 80% (studies summarized in Knight et al. [1]). However, the mating status of wild females trapped in these orchards suggested CM MD reduces mating by <20% [2,3].

Several behavioral factors have been identified to affect female mating in MD-treated orchards, such as pheromone autodetection, dispersal, and delayed mating [1]. Unfortunately, the importance of MD impacting these female behaviors cannot be easily quantified in field trials and remains unclear. A new four-component dual-sex lure (PHEROCON^®^ MEGALURE CM DUAL 4K, Trécé Inc., Adair, OK, USA) now makes it much easier to sample female CM and measure mating status [4].

A recent two-year survey of 142 CM populations in orchards treated with CM MD found that the proportion of mating of females caught in dual-sex lure-baited delta traps ranges from <0.20 to >0.80 [3]. No differences were found in apple versus pear. Populations in organic versus conventionally managed orchards had significantly higher proportions of mated females in one of the two years. In general, a higher proportion of CM females were mated in the second half of the season (2nd and 3rd flights). Interestingly, no difference was found among six different CM MD programs, including aerosols and several hand-applied dispensers used at 2.5–1000 units ha^−1^. The mean proportion of unmated females across both years under CM MD was 0.43 compared with 0.20 in untreated orchards. One significant exception occurred when an organic apple grower accidentally combined the use of aerosol units with low rates of hand-applied dispensers in four orchards. The proportion of unmated females caught in these orchards was >0.80 over the entire season. Finally, a few conventional orchards treated with MD plus conventional insecticide sprays had similar, high levels of unmated females [3].

The success of management strategies based on reducing CM oviposition by disrupting mating of wild females, such as MD or releases of sterilized moths, requires a relatively low level of moth immigration, especially a very low number of wild mated females [5]. Several orchards where the mating status of females was assessed in 2021–2022 were identified post hoc as likely experiencing significant levels of immigration [3]. These orchards were categorized based on high moth catches and variable levels of fruit injury, despite low catches and the absence of injury the previous year. In a few cases, neighboring pockets of poorly managed or unmanaged hosts were found within 500 m. In other orchards, there was no explanation for this unexpected increase in CM pressure. We hypothesized that these situations may be from CM immigrants from unknown and more distant hosts. Mark–recapture studies have found that CM adults can fly up to 10 km [6,7]. Both mated and unmated male and female CMs are similarly dispersive [8]. Thus, differential immigration rates into CM MD-treated orchards limits an accurate assessment of the effectiveness of specific MD programs.

Standardized mark–recapture methods inside MD-treated orchards have been proposed as an approach to minimize the impacts of differential immigration [3]. Weekly releases of marked sterilized male and female CMs with a low proportion of mating could allow various CM MD programs and the impact of pesticide sprays to be assessed within selected orchards and management programs. This approach has been used to assess the effectiveness of a microencapsulated (MEC) formulation of (*E*,*E*)-8,10-dodecadien-1-ol (codlemone) (CIDETRAK^®^ CM MEC^TM^, Trécé Inc., Adair, OK, USA) with hand-applied dispensers in two field trials [9]. The combined programs had significantly higher proportions (2- to 3-fold) of unmated wild and sterilized female CMs than the dispenser-only programs in one of the two years. Based on these initial results, we report here more extensive studies of single and combined CM MD programs. Eleven trials using releases of sterilized CM adults were conducted in 2023–2024 in 82 orchards to assess 20 programs, including both individual and combination CM MD programs and comparative untreated orchards.

## 2. Materials and Methods

### 2.1. Lures and Traps

A proprietary binary lure was used in this study to sample male and female CMs (PHEROCON^®^ MEGALURE CM DUAL 4K lure, Trécé Inc., Adair, OK, USA; hereafter named as ‘CM4K’). A black PVC matrix loaded with pear ester ((*E*,*Z*)-2,4-ethyl decadienoate), DMNT ((*E*)-4,8-dimethyl-1,3,7-nonatriene), and pyranoid linalool oxide (6-ethenyl-2,2,6-trimethyloxan-3-ol) was used in combination with a white membrane dispenser loaded with acetic acid. Orange delta traps (PHEROCON^®^ DELTA VI, Trécé Inc., Adair, OK, USA) were attached to poles and placed in the canopy at ca. 3.0 m. Five traps were spaced ≥25 m apart and >20 m from the borders of the plots down a single row in the middle of the plot. Lures were placed in the center of the sticky liner (CleanBrake^®^, Trécé Inc., Adair, OK, USA). Lures were replaced after 8 weeks, and liners were replaced every two weeks unless no moths were caught. Moths on liners were counted and sexed in the laboratory. The female mating status was determined by dissecting the *bursa copulatrix* for the presence of spermatophores using a stereomicroscope.

### 2.2. MD Dispensers

Orchards were either left untreated or treated with MD dispensers for CM. Ten types of MD dispensers were included in the various studies (Table 1) and used at variable rates according to manufacturers’ recommendations [10]. Specifically, two aerosol formulations, six hand-applied dispensers and two microencapsulated sprayable formulations (MEC) were considered. Dispensers contained either codlemone alone or a blend of codlemone and pear ester, and a formulation also included the sex pheromone blend of *Grapholita molesta* (Busck) and Oriental fruit moth (OFM), comprising (*Z*)-8-dodecen-1-yl acetate, (*E*)-8-dodecen-1-yl acetate, and (*Z*)-8-dodecen-1-ol (ratio 90.4:8.2:1.4). In addition to the commercial products, a new experimental codlemone/pear ester dispenser type, CPD (Concentrated Passive Dispenser), was constructed as a cluster of 8 CIDETRAK^®^ CMDA COMBO MESO-A dispensers clipped onto a 15 cm circular plastic hanger and applied at 10 units ha^−1^, providing the same total amount of codlemone and pear ester per hectare as with the CIDETRAK^®^ CMDA COMBO MESO-A applied at 80 dispensers ha^−1^ (Trécé Inc., Adair, OK, USA) (Table 1).

All MEC sprays and MD dispensers except for the CPD were applied by the growers. MEC sprays were applied with an air blast sprayer on four dates beginning mid-May and repeated on a 4–5-week schedule until mid-August. Aerosols were placed in a 64 m × 64 m grid starting 25 m from the edge of the orchards. CPD dispensers were placed in a 31 m × 31 m grid. Placement of hand-applied dispensers typically began <5 m from the edge of the block and were applied on every row at a spacing of 2 m to 3 m depending on their tree density, canopy closure, and row spacing.

### 2.3. Trials Description

Studies were conducted within 82 commercial apple and pear orchards located in the Yakima Valley in Washington State, USA during the two-year project (2023–2024). Orchards included all the major cultivars of both apple (i.e., Red Delicious, Golden Delicious, Honeycrisp, Gala, Fuji, Ambrosia, Granny Smith, Envy, Cripps Pink, and Jazz) and pear (i.e., Bartlett, Red Bartlett, D’Anjou, Red Anjou, and Bosc) grown in the area, and ranged from central leader to high-density trellis blocks. Orchard size ranged from 2.0 to 10 ha. All orchards were sprayed by growers with standard management and nutritional programs. Conventional sprays applied for CM varied from 1 to 5 applications of diamide, spinosyn, and neonicotinoid chemistries. Organic blocks were treated with multiple applications of granulosis virus, horticultural oil, and 0–4 sprays of a spinosyn insecticide. A total of eleven trials were conducted considering either individual or combined MD programs, as described hereafter.

#### 2.3.1. Individual CM MD Programs

Four or five orchards were left untreated with MD each year and were included as a control during each trial period. Four studies were conducted during 2023 (Trials 1–4) and one in 2024 (Trial 5) to compare the mating of sterile and wild female CM under a single MD program. Trial 1 tested NoMate CM in five orchards over a three-week period beginning on 29 May. Trial 2 evaluated Isomate CM Mist for three weeks in five orchards beginning on 26 June. Trial 3 evaluated CIDETRAK^®^ CMDA COMBO MESO-A in six orchards for two weeks beginning on 28 June. Trial 4 evaluated Isomate Flex CM for six weeks in five orchards beginning on 24 July. Finally, Trial 5 conducted in 2024 evaluated two single component CM MD programs, the experimental CPD and CIDETRAK^®^ CMDA COMBO PP, in 2 to 4 orchards for 16 weeks beginning on 13 May.

#### 2.3.2. Multi-Component CM MD Programs

Compound CM MD programs were evaluated in both years including aerosols, three hand-applied dispensers, and the two sprayable formulations. Two combined CM MD programs were evaluated during 2023 (Trials 6–7). Trial 6 compared CIDETRAK^®^ CMDA COMBO PP against CIDETRAK^®^ CMDA COMBO PP plus Isomate CM Mist in five orchards for 10 weeks beginning on 1 May. Trial 7 evaluated NoMate CM plus Isomate CM Mist in four orchards from 10 July for five weeks. Several combination CM MD programs were grouped into Trials 8–9 and evaluated in 2024 for 16 weeks beginning on 6 May. Trial 10 conducted during 2024 included CIDETRAK^®^ CMDA + OFM MESO plus CIDETRAK^®^ DA MEC and CIDETRAK^®^ CMDA COMBO PP dispensers starting 1 May for 10 weeks. Finally, Trial 11, conducted during 2024, included two additional MD treatments that were monitored but not replicated. This included the use of Semios CM Eco Aerosol and CIDETRAK^®^ CMDA COMBO MESO-A plus the Semios CM Eco Aerosol evaluated for 8 weeks beginning on 1 May.

### 2.4. Sterilized Moth Releases

Sterilized laboratory-reared and unsexed *C. pomonella* adults were obtained from M3 Agriculture Technologies (Dayton, OH, USA). Moths were reared and sterilized at the Okanagan–Kootenay Sterile Insect Release Program’s mass-rearing facility (Osoyoos, BC, Canada). Sterilized moths are marked with an internal red dye which allows them to be easily differentiated from wild moths.

Sterilized moths were collected in Canada on Friday and Saturday within cardboard cups (3200 moths per cup), chilled, and shipped to Washington State. Moths were picked up on Monday mornings between 1000 and 1100 h and were estimated to have been chilled for 60 h after collection and sterilization. Moths were placed in a chilled cooler and driven from a central location using an all-terrain vehicle over gravel paths and through orchard grass-covered drive lanes over a 2–3 h period. Sterile moths were tapped on the ground at a rate of 800 moths per 180 m down the trap row. The goal was to release ca. 160 moths within 20 m on either side of each of the five traps in the row.

Moths were released weekly from 1 May until 11 September in 2023 and from 6 May to 26 August in 2024. Moths, due to technical issues, were not available on three and two weeks in each year, respectively. Considering an average sex ratio 1:1, the recapture rates of sterile female CMs ranged from 2.6% to 6.1%, with an average value of 4.1% across trials. Recapture rates of sterile CMs were sufficient to obtain a reliable number of females to be dissected per each treatment and replicate of this study.

In both years, before each weekly release, samples of 30 female moths were collected, frozen, and later dissected to determine their mating status prior to the release. The proportion of mated sterile moths in the weekly releases varied from 0.00 to 0.25 in both years, with a mean value ± standard error (SE) of 0.12 ± 0.02 in 2023 and 0.09 ± 0.01 in 2024. The released and recollected sterile female moths were dissected to determine their mating status after their exposure to different CM MD programs in the various trials. The proportions of field-recollected mated sterile females were corrected for the initial proportions of mated sterile females released.

### 2.5. Fruit Injury Assessment, 2024

During the second year of the study, 28 apple orchards treated with CM MD were sampled for percent fruit injury from CMs. Samples were conducted during the last week of August. Thirty to forty sampling spots situated within 30 m from the center of the trap transect row were arbitrarily picked. Samples included 60 visually inspected fruits (1800–2400 fruits per sample) between 2.0 and 3.5 m in the canopy.

### 2.6. Statistical Analysis

Statistical analyses of the moth catches and the level of fruit injury were performed with R software v. 4.0.3 [11]. Data were analyzed according to their distribution. The proportion of unmated sterile females was corrected with Schneider-Orelli’s formula based on the proportion previously mated at the rearing facility. Data normality was tested with Shapiro–Wilk’s test and Levene’s Test for homogeneity of variance. A linear model (LM) was used with normal data, while data found to be close to a normal distribution were transformed using the square root function (sqrt(x+0.5)). Data which were not normally distributed fit a Poisson distribution and a generalized linear model (GLM) was used. Akaike’s information criteria (AIC) and residuals were both used to select the fitted models in each analysis. A multiple comparison post hoc test was performed on the fitted models (GLHT function from multcomp package) and Tukey’s HSD test (*p* < 0.05) was used to discriminate significant differences among treatments. Treatments with a mean value of zero were not included in analyses. Mating status data from each site were used in the analyses only when a sample ≥ 5 CM females was dissected. Data are reported as mean values ± standard error (SE).

## 3. Results

### 3.1. Individual CM MD Programs

Four short-term studies were conducted to assess the proportion of mated female CMs under individual CM MD programs in 2023 (Table 2). No differences in mating were found between either NoMate CMs or Isomate CM Flex and the untreated control with either wild or sterile moths. In contrast, the proportions of unmated female CMs were higher for both wild and sterile moths in the CIDETRAK^®^ CMDA COMBO MESO-A program than in the control. Similarly, in the Isomate CM Mist program, the unmated sterile females were higher than in the control, while no wild moth was recorded in these orchards (Table 2). In all these trials, where wild CMs were recorded, there was no difference in the proportions of unmated sterile versus wild female moths. 

Two individual CM MD programs were evaluated in 2024: the experimental CPD and CIDETRAK^®^ CMDA COMBO PP dispensers (Table 2). Only the experimental CPD program had a significantly greater proportion of unmated females than the untreated control. Also, the proportions of unmated sterile female moths were greater than for wild moths across treatments.

### 3.2. Multi-Component CM MD Programs

Studies during 2023 evaluated three multi-component CM MD programs (Table 3). Trial 6 found that the CIDETRAK^®^ CMDA COMBO PP plus Isomate CM Mist program had a significantly higher proportion of unmated females than the individual CIDETRAK^®^ CMDA COMBO PP program. This single CM MD program did not have a lower proportion of unmated females than the control. The proportion of unmated sterile females was significantly greater than with wild females across all three treatments. Trial 7 evaluated a NoMate CM dispenser plus Isomate CM Mist aerosol program versus untreated orchards, and the level of unmated sterile females was found to be higher in the combined MD program than in the control. On the contrary, no difference in female mating status was recorded for wild moths. The interaction of moth type and MD treatment was significant because the proportion of unmated sterile moths was different between treatments but not for wild moths (Table 3).

Two large studies comparing multiple programs were conducted in 2024 (Table 4). Both studies used the combination of CIDETRAK^®^ CMDA COMBO PP plus CIDETRAK^®^ CM MEC and CIDETRAK^®^ DA MEC as a positive control. Trial 8 considered the effectiveness of reducing the density of the CIDETRAK^®^ CMDA COMBO PP to 500 ha^−1^ in combination with the combined use of sprayable microencapsulated codlemone and pear ester applications (MEC formulations). Trial 9 looked at these dispensers applied at 840 ha^−1^ in combination with the Isomate CM Mist aerosol. The proportions of unmated sterile female CMs were significantly higher than for wild female moths across treatments in both studies. The highest proportion of unmated sterile female CMs in Trial 8 was with CIDETRAK^®^ CMDA COMBO PP at the low dispenser rate (500 ha^−1^) combined with sprayable codlemone (0.93). Similarly, the highest proportion of unmated sterile moths in Trial 9 was with the reduced dispenser rate of CIDETRAK^®^ CMDA COMBO PP (840 ha^−1^) in combination with the aerosol and pear ester sprays (0.95). Intermediate proportions of unmated female CMs in Trial 8 occurred with the combined CIDETRAK^®^ CM MEC and CIDETRAK^®^ DA MEC sprays and the full rate of CIDETRAK^®^ CMDA COMBO PP (1000 ha^−1^) combined with either codlemone/pear ester sprays or with the aerosol (Table 4).

A third study (Trial 10) was conducted until mid-season in 11 apple orchards. Both CM MD programs had significantly higher proportions of unmated female CMs than in untreated orchards (Table 4). The combined program with CIDETRAK^®^ CMDA + OFM MESO dispensers plus CIDETRAK^®^ DA MEC sprays had a mean proportion of unmated CM females 2.5 times greater than using only CIDETRAK^®^ CMDA COMBO PP dispensers (Table 4). Two other CM MD programs were evaluated in single orchards in Trial 11. The proportions of unmated female CMs were 0.31 and 0.77 in orchards treated with either the Semios CM Eco Aerosol or the same aerosol formulation in combination with the CIDETRAK^®^ CMDA COMBO MESO-A dispensers, respectively.

### 3.3. Wild and Sterile CM Females Mating Status

The effectiveness of assessing female CM mating with wild versus sterilized moths was conducted across 74 MD orchard treatments, where ≥5 wild females were dissected. This included the dissections of 3358 wild and 5659 sterile female moths. The mean numbers of wild and sterile females dissected per orchard treatment were 34.8 ± 5.3 and 58.1 ± 9.2, respectively. The overall proportions of unmated female CMs were 0.41 ± 0.02 and 0.59 ± 0.02 for the wild and sterile females, respectively. The mean difference in the proportion of unmated females, 0.18 ± 0.02 (95% CL = 0.15–0.22), was significantly higher with the sterile than wild females (paired *t*-test: t = 9.68, *p* < 0.0001). We were unable to collect > 5 wild females from 21 orchards during the two-year study.

**Figure 1:**
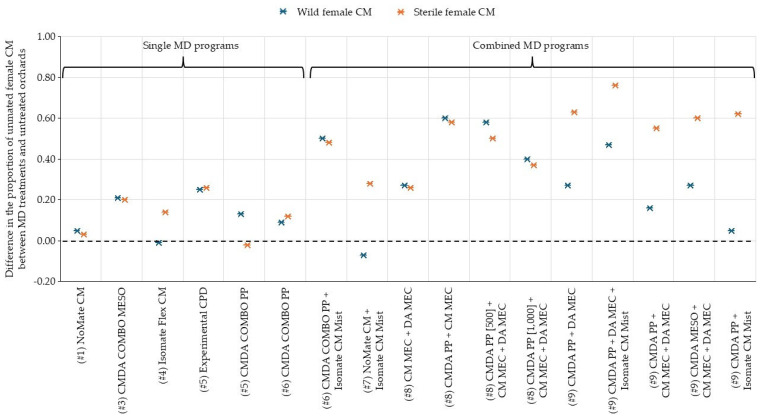
Summary of the difference in the proportions of wild and sterile female CMs across 6 programs using a single MD tactic and 11 programs using a combination MD tactic, expressed as the difference with the untreated plots of the same trial, in 2023–2024. The trial number is reported within round brackets ( ), while for similar treatments the MD application rate is specified within square brackets [ ].

The difference in the proportion of the unmated female CMs between MD treatments and untreated plots varied between wild and sterile moths across this study (Figure 1). The combined MD programs recorded the highest differences (ranging between +0.26 and +0.76 for sterile CMs) compared to single MD programs (maximum +0.26 for sterile CMs).

### 3.4. Level of Fruit Injury, 2024

There were 28 apple orchards in 2024 where we were able to measure the female mating status of both wild and sterile female CMs (Table 5). Among this group, 17 orchards had detectable levels of fruit injury ranging from 0.1 to 2.6%, mean = 1.14 ± 0.23. Twelve of these orchards were farmed organically and eight orchards were known to be near unmanaged sources of CMs. Both management and proximity to unmanaged hosts were significant factors affecting fruit injury. The interaction between management and immigration was not significant. Differences in the proportions of unmated female CMs between wild and sterilized moths were significant in orchards with injury, orchards with or without nearby sources of unmanaged CMs, and in organically farmed orchards. The proportion of unmated female CMs between wild and sterilized moths was not different in orchards without fruit injury and in conventional orchards (Table 5).

## 4. Discussion and Conclusions

A series of studies begun in 2021 has used the CM4K lure to measure the mating status of female CM populations in MD-treated orchards in the Pacific Northwest, USA [3]. The annual data collected over this four-year period have been largely consistent. Each year, we have found there is a large variability in the proportions of unmated female CMs across MD-treated orchards, regardless of the individual MD program deployed. The yearly mean proportion of unmated wild female CMs sampled has consistently been <0.50. In sharp contrast, an early and serendipitous study in 2021 found that the proportion of unmated females can be significantly increased in orchards treated with two concurrent MD programs (aerosols plus hand-applied codlemone/pear ester dispensers). Studies conducted with combination programs of codlemone/pear ester dispensers with periodic spray applications of sprayable microencapsulated codlemone demonstrated similar results [9]. This initial work was widely expanded in our current study to include 20 different CM MD programs.

The highest levels of mating disruption of wild female CMs in 2023–2024 were found with mixed programs combining sprayable pear ester, sprayable codlemone, codlemone/pear ester passive dispensers, and codlemone aerosols. Mean proportions of unmated sterile female CMs were >0.70 and as high as 0.95. These data were consistent with the concurrent studies combining sprayable codlemone with codlemone/pear ester dispensers [9].

The additional use of sprayable pear ester for CM MD did not demonstrate a noticeable effect in five treatments, including sprayable codlemone and two programs with codlemone/pear ester passive dispensers or codlemone aerosols. A previous study found that the combination of sprayable codlemone and sprayable pear ester significantly reduced male catches, but not female mating proportions, compared with either formulation used alone at low or high spray volumes [12]. However, one study suggested sprayable pear ester has a negative effect on CM mating, such as reducing the proportions of multiple-mated females [13]. Nevertheless, the use of CIDETRAK^®^ DA MEC as a spray adjuvant with granulosis virus in organic orchards or with the anthranilic diamide chlorantraniliprole in conventional orchards is effective in reducing levels of fruit/nut injury [14,15,16,17].

Chlorantraniliprole has exhibited a strong negative effect on CM female mating [18]. Across the four years, a variable number of chlorantraniliprole sprays (0–3) were applied to the conventional orchards during the first summer larval generation. Thus, establishing the contributory effect of chlorantraniliprole on female CM mating in variable CM MD and spray programs will require a more defined protocol than used here.

The use of mark–recapture techniques with releases of sterilized male and female CM adults under the various CM MD programs improved our field assessments of CM MD programs in three ways. First, the weekly releases of sterile moths according to our protocol allowed significantly more CM females to be sampled. Larger sample sizes have likely improved the precision of our estimated means. The higher CM catches also allowed us to exclude fewer orchards from our analyses, despite the concurrent low catch of wild females (those catching <5 CM females). Third, in orchards where sizeable resident or immigrant wild CM populations occurred, data obtained from the sterile moths provided a more realistic measure of the CM MD technology, but not necessarily its level of effectiveness. A final benefit is that the potential exposure of female moths to sublethal insecticide residues could be less significant with the released sterile versus resident wild females.

The threat of mated females immigrating into MD-treated orchards has always presented a dire warning to growers implementing an unbalanced reliance on this management strategy [19]. Flight mills have demonstrated mated and unmated male and female CMs have the potential for similar long-distance flights [8]. However, the mating status of female CM immigrants has not been measured in the field. Clearly, a sizable movement of previously mated female CMs can stymie the effective use of either MD or sterile moth releases [4]. Studies demonstrating higher levels of fruit injury or moth catches along orchard’s borders can reflect either immigration or a pooling of moths from the interior of the orchard if moths are reticent to leave the canopy structure [20,21]. Thus, managers are uncertain if their pest problem is interior or exterior.

Areawide management programs for CMs were successful and clearly jump-started the adoption of CM MD [22,23,24]. The effectiveness of these programs was derived from a combination of enhanced intra-grower communication, using effective monitoring and management tools, and removing poorly managed hosts out to boundaries not impacted by immigrant moths [22]. It would be interesting, now that we have a useful tool (CM4K lure), to monitor female CMs to map levels of mating within these large sites, and using the sterile moth protocol outlined here would likely be an effective approach. Establishing whether male catch shutdown in traps as practiced today is a reliable measure of CM MD could be a great benefit.

Data collected over four years (2021–2024) from 110 different apple and pear orchards (>200 orchard-year combinations) suggest that the typical use of CM MD by growers is insufficient in reducing female mating [3]. There is a lack of clarity on how CM MD has been adopted on 100,000s ha, but the fact that research cannot demonstrate that an effective level of disruption of female mating occurs is a conundrum. The prerequisite of CM MD working best, that of pest population densities being low, is commonly cited as a major prerequisite for MD to be effective [25]. Yet, there is no concrete evidence that the disruption of mating is greater when the adult population density is lower. In fact, little difference was found in the proportion of mated female CMs despite a 20-fold range in the catch of female moths from different orchards when using a pear ester lure [26]. Unfortunately, our current study with the CM4K lure was not structured to evaluate the relation between population density and female mating.

Our four years of study have established that using CM MD can decrease mating, on average, by 20%, and that using a 2X rate of CM MD can significantly reduce mating by an additional 35%. A benefit–cost analysis of increasing the effectiveness and cost of CM MD strongly depends on the crop’s value. Conventional growers carefully monitoring their orchard can always use more insecticides or spray borders without increasing MD rates. Organic growers farming the most valuable fruit and already spraying throughout the season would be the most interested in increasing the rates of CM MD, if they believed it was effective. The limited studies examining higher rates of CM MD have not been interpreted as being more effective, yet active measures of female mating success have not been used [9]. Increased dispenser rates have largely been restricted to the orchard’s borders, and any added value from this action has not been meaningfully assessed [3].

The release of hundreds of sterile males and females along a relatively short section of an orchard row could impact the proportions of wild and sterile females found mated. The quality of the sterilized moths released is a key factor influencing mating. Moths being released were often in a rough state (loss of wing scales) due to experiencing 60 h of cold storage and transport along dirt and gravel paths to the orchard. The estimated recapture rate of sterile moths was only <5% over the project. Sterile CMs are reared at a constant high temperature in the Canadian laboratory and compete poorly with wild moths in the field, especially when temperatures are cool [27,28,29]. Adopting a standard protocol throughout our extensive trials is thought to reflect true differences among CM MD programs. In partial support, the proportions of unmated wild females measured across orchards treated with a single CM MD program was similar to our earlier study without sterile moth releases [3].

Dual MD for CMs and OFMs in pome fruit has included dispensers, sprayables, attracticides, and aerosols [30,31,32,33,34]. The success of these programs has largely been assessed via male moth catch reductions in sex pheromone-baited traps and effective crop protection. Interestingly, the impact of dual CM/OFM MD versus the use of individual MD for each program on female CM mating has not been reported. However, in 2024, the mean proportion of unmated females in two orchards treated with the CM/OFM MD dispenser plus pear ester sprays was unexpectedly high, 0.79, especially when compared with nearby orchards treated only with codlemone/pear ester dispensers for CM, 0.32. Our previous study did not find that codlemone/pear ester MESO dispensers for CMs are more effective than other codlemone/pear ester dispenser types. Also, in the 2021 study, with the combined use of MESO dispensers plus aerosols, which was our initial evidence that increasing the current level of CM MD can significantly lower female mating, the four orchards were also treated with aerosol units for both OFMs and the oblique banded leafroller, *Choristoneura rosaceana* (Harris) [3].

Several studies have examined male and female catches in traps combining CM and OFM pheromone lures, often in combination with acetic acid and terpinyl acetate co-lures. Inclusion of the three-component OFM sex pheromone largely shuts down CM catch, but the reverse situation does not occur [35]. This negative effect of OFM sex pheromone on CM catch was overcome with the addition of a single UV-A LED in the trap [36]. Thus, both CM sexes were attracted close to the traps but only lured inside by a proximity to the dim UV-A light [37]. We hypothesize from our two studies that the OFM pheromone released at the high rates from MD dispensers or aerosols may impact mating of female CMs. Further studies should examine the behavioral impacts of OFM pheromone on male and female CM sexual behaviors. Co-application of an OFM sprayable formulation with one or more CM MD programs should likely be the first evaluation.

## Figures and Tables

**Table 1:** Summary of CM MD dispensers used in growers’ orchards, 2023–2024.

Type of CM MD	Manufacturer	Active Ingredients Loading	Dispenser or Liquid Rate ha^−1^
Semios CM Eco Aerosol	Semios BIO Technologies Inc., Vancouver, BC, Canada	55.4 g codlemone	2.5
Isomate CM Mist	Pacific Biocontrol Corp., Vancouver, WA, USA	23.6 g codlemone	2.5
NoMate CM Spiral	Scentry Biologicals Inc., Billings, MT, USA	135 g codlemone	1000
Isomate CM Flex	Pacific Biocontrol Corp., Vancouver, WA, USA	95.5 g codlemone + 62 mg of 1-dodecanol and 1-tetradecanol	1000
CIDETRAK CMDA COMBO PP	Trécé Inc., Adair, OK, USA	90 g codlemone 60 g pear ester	500–1000
CIDETRAK CMDA COMBO MESO-A	Trécé Inc., Adair, OK, USA	850 g codlemone 500 g pear ester	80
Experimental CPD	Trécé Inc., Adair, OK, USA	850 g codlemone 500 g pear ester	10
CIDETRAK CMDA + OFM MESO	Trécé Inc., Adair, OK, USA	800 mg codlemone + 450 mg pear ester + 550 mg OFM PH blend	80
CIDETRAK CM MEC	Trécé Inc., Adair, OK, USA	7% codlemone	250 mL
CIDETRAK DA MEC	Trécé Inc., Adair, OK, USA	5% pear ester	30 mL

**Table 2:** Summary of the proportions of wild and sterile unmated codling moth females trapped within various programs of mating disruption versus untreated orchards during 2- to 12-week periods in Trials 1–5 (N = 4–6), 2023–2024.

Trial #, Dates, # Replicates	Treatments ^b^	Mean ± SE Proportion of Unmated Females ^a^	
		Wild [# Females]	Sterile [# Females] ^c^
1, 29 May–19 June 2023, N = 5	Untreated	0.24 ± 0.09 Aa [22]	0.40 ± 0.09 Aa [201]
	NoMate CM	0.29 ± 0.05 Aa [211]	0.43 ± 0.04 Aa [250]
		Treatments: F_1,16_ = 0.58, *p* = 0.4572 Moths: F_1,16_ = 2.35, *p* = 0.1446 Treatments × moths: F_1,16_ = 0.18, *p* = 0.6742	
		
2, 26 June–18 July 2023, N = 5	Untreated	-	0.39 ± 0.07 A [243]
	Isomate CM Mist	-	0.64 ± 0.02 B [366]
		Treatments: F_1,8_ = 6.29, *p* = 0.0080	
		
3, 28 June–12 July 2023, N = 6	Untreated	0.11 ± 0.05 Aa [30]	0.25 ± 0.08 Aa [89]
	CIDETRAK CMDA COMBO MESO-A	0.32 ± 0.03 Ba [109]	0.45 ± 0.10 Ba [45]
		Treatments: F_1,20_ = 6.97, *p* = 0.0157 Moths: F_1,20_ = 2.96, *p* = 0.1009 Treatments × moths: F_1,20_ = 0.73, *p* = 0.4026	
		
4, 24 July–4 September 2023, N = 5	Untreated	0.22 ± 0.05 Aa [32]	0.24 ± 0.06 Aa [166]
	Isomate Flex CM	0.21 ± 0.02 Aa [97]	0.38 ± 0.04 Aa [94]
		Treatments: F_1,16_ = 2.10, *p* = 0.1670 Moths: F_1,16_ = 3.84, *p* = 0.0677 Treatments × moths: F_1,16_ = 2.14, *p* = 0.1625	
		
5, 13 May–7 September 2024, N = 4	Untreated	0.19 ± 0.06 Bb [105]	0.35 ± 0.01 Bb [955]
	Experimental CPD	0.44 ± 0.03 Aa [155]	0.61 ± 0.02 Aa [854]
	CIDETRAK CMDA COMBO PP	0.32 ± 0.05 Bb [122]	0.33 ± 0.08 Bb [165]
		Treatments: F_2,18_ = 13.91, *p* = 0.0002 Moths: F_1,18_ = 7.76, *p* = 0.0122 Treatments × moths: F_2,18_ = 1.71, *p* = 0.2093	

^a^ Means followed by a different uppercase letter were significantly different across treatments and lowercase letters between moth types, *p* < 0.05, Tukey’s test.

^b^ Isomate CM Flex and NoMate CM dispensers were applied at 1000 units ha^−1^. CIDETRAK^®^ CMDA COMBO PP and CIDETRAK^®^ CMDA COMBO MESO-A were applied at 840 and 80 units ha^−1^, respectively. CPD was applied at 10 units ha^−1^. Isomate CM Mist aerosol was applied at 2.5 units ha^−1^.

^c^ Means for the proportion of mated sterile females [number of females dissected] were corrected for the initial proportion of mated sterile females released across the 17 weeks of the 2023 season, mean = 0.12 ± 0.02 [509]; and 16 weeks in 2024, mean = 0.09 ± 0.01 [480] (Henderson-Tilton’s formula).

**Table 3:** Summary of the proportions of wild and sterile unmated codling moth females trapped within various combination programs of mating disruption versus untreated orchards during 4- to 10-week periods in Trials 6–7 (N = 4–5), 2023.

Trial #, Dates, Replicates	Treatments ^b^	Mean ± SE Proportion of Unmated Females ^a^	
		Wild [# Females]	Sterile [# Females] ^c^
6, 1 May–10 July 2023, N = 5	Untreated	0.21 ± 0.06 Aa [45]	0.35 ± 0.05 Ab [786]
	CIDETRAK CMDA COMBO PP	0.30 ± 0.06 Aa [245]	0.47 ± 0.06 Ab [427]
	CIDETRAK CMDA COMBO PP + Isomate CM Mist	0.71 ± 0.09 Ba [52]	0.83 ± 0.05 Bb [348]
		Treatments: F_2,24_ = 26.66, *p* = 0.0001 Moths: F_2,24_ = 5.46, *p* = 0.0282 Treatments × moths: F_2,24_ = 0.08, *p* = 0.9190	
		
7, 10 July–10 August 2023, N = 4	Untreated	0.34 ± 0.09 Aa [36]	0.34 ± 0.03 Aa [198]
	NoMate CM + Isomate CM Mist	0.27 ± 0.05 Aa [142]	0.62 ± 0.03 Ba [288]
		Treatments: F_1,12_ = 2.21, *p* = 0.1633 Moths: F_1,12_ = 4.12, *p* = 0.0651 Treatments × moths: F_1,12_ = 6.77, *p* = 0.0232	

^a^ Means followed by a different uppercase letter were significantly different across treatments and lowercase letters between moth types, *p* < 0.05, Tukey’s test.

^b^ NoMate CM dispensers were applied at 1000 units ha^−1^. CIDETRAK^®^ CMDA COMBO PP dispensers were applied at 840 units ha^−1^. Isomate CM Mist aerosol was applied at 2.5 units ha^−1^.

^c^ Means for the proportion of mated sterile females [number of females dissected] were corrected for the initial proportion of mated sterile females released across the 17 weeks of the 2023 season, mean = 0.12 ± 0.02 [509] (Henderson-Tilton’s formula).

**Table 4:** Comparison of the proportions of unmated wild and released sterilized female moths trapped under codling moth mating disruption programs, including both single and combined programs evaluated during 2024, in Yakima Valley, Washington.

Trial #, Dates	Treatments ^b^	Number of Replicates	Mean ± SE Proportion of Unmated Females ^a^	
			Wild [# Females]	Sterile [# Females] ^c^
8, 6 May–26 August 2024	Untreated	4	0.17 ± 0.07 [105] Cb	0.35 ± 0.01 [955] Ca
	CM MEC + DA MEC	4	0.44 ± 0.03 [155] Bb	0.61 ± 0.02 [854] Ba
	CMDA PP [500] + CM MEC	2	0.77 ± 0.03 [130] Ab	0.93 ± 0.07 [91] Ab
	CMDA PP [500] + CM MEC + DA MEC	2	0.75 ± 0.15 [66] Ab	0.85 ± 0.03 [110] Aa
	CMDA PP [1000] + CM MEC + DA MEC	2	0.57 ± 0.03 [285] ABb	0.72 ± 0.00 [47] ABa
			Treatments: F_4,17_ = 20.95, *p* < 0.0001 Moths: F_1,17_ = 9.78, *p* = 0.0061 Treatments × moths: F_4,17_ = 0.36, *p* = 0.8361	
			
9, 6 May–26 August 2024	Untreated	4	0.32 ± 0.11 [30] Cb	0.19 ± 0.06 [198] Ca
	CMDA PP [840] + DA MEC	5/6	0.59 ± 0.06 [480] ABb	0.82 ± 0.04 [100] ABa
	CMDA PP [840] + DA MEC + Isomate CM Mist	4	0.79 ± 0.07 [155] Ab	0.95 ± 0.02 [66] Aa
	CMDA PP [1000] + CM MEC + DA MEC	3	0.48 ± 0.03 [368] Bb	0.74 ± 0.03 [277] Ba
	CMDA MESO-A + CM MEC + DA MEC	3	0.59 ± 0.06 [216] ABb	0.79 ± 0.13 [84] ABa
	CMDA PP [1000] + Isomate CM Mist	2	0.37 ± 0.04 [114] Bb	0.81 ± 0.08 [34] Ba
			Treatments: F_5,31_ = 13.97, *p* < 0.0001 Moths: F_1,31_ = 14.24, *p* = 0.0007 Treatments × moths: F_5,31_ = 2.14, *p* = 0.0870	
			
10, 1 May–10 July 2024	Untreated	3	0.17 ± 0.03 [119] C	-
	CMDA/OFM MESO + DA MEC	2	0.79 ± 0.10 [49] A	-
	CMDA PP [1000]	6	0.32 ± 0.01 [371] B	-
			Treatments: F_2,8_ = 42.35. *p* < 0.0001	

^a^ Means followed by a different uppercase letter were significantly different across treatments and lowercase letters between moth types, *p* < 0.05, Tukey’s test.

^b^ CIDETRAK^®^ CM MEC (CM MEC) and CIDETRAK^®^ DA MEC (DA MEC) were applied at 250 and 30 mL ha^−1^, respectively, four times a season every 4–6 weeks. CIDETRAK^®^ CMDA COMBO PP (CMDA PP) was applied at 500–840–1000 units ha^−1^ according to the treatment. CIDETRAK^®^ CMDA COMBO MESO-A (CMDA MESO-A) and CIDETRAK^®^ CMDA + OFM MESO (CMDA/OFM MESO) were applied at 80 units ha^−1^. Isomate CM Mist aerosol was applied at 2.5 units ha^−1^.

^c^ Means for the proportion of mated sterile females [number of females dissected] were corrected for the initial proportion of mated sterile females released across the 16 weeks in 2024, mean = 0.09 ± 0.01 [480] (Henderson-Tilton’s formula).

**Table 5:** Summary of the mean fruit injury and the proportions of unmated wild and sterilized female CMs among orchards categorized with or without injury, with or without nearby sources of unmanaged CM populations, and in orchards farmed organically or conventionally, N = 28, 2024.

Comparison	Description	Number of Orchards	Mean ± SE Percent Fruit Injury ^a^	Mean ± SE Proportion Unmated Females ^b^		Paired *t*-Test
				Wild	Sterile	
Injury	With	17	1.14 ± 0.23	0.46 ± 0.03 B	0.64 ± 0.04 A	*t*_16_ = 3.81, *p* = 0.0016
	None	11	0.00 ± 0.00	0.58 ± 0.04 A	0.65 ± 0.03 A	*t*_10_ = 1.40, *p* = 0.1900
						
Immigration	Known	8	1.41 ± 0.25 a	0.42 ± 0.04 B	0.59 ± 0.05 A	*t*_7_ = 2.48, *p* = 0.0419
	Unknown	20	0.41 ± 0.19 b	0.55 ± 0.03 B	0.67 ± 0.03 A	*t*_19_ = 2.87, *p* = 0.0099
						
Management	Organic	12	1.16 ± 0.29 a	0.42 ± 0.03 B	0.70 ± 0.04 A	*t*_11_ = 7.61, *p* < 0.0001
	Conventional	16	0.35 ± 0.17 b	0.58 ± 0.03 A	0.60 ± 0.03 A	*t*_15_ = 0.64, *p* = 0.5287
Analysis for fruit injury: Immigration Management Immigration × management			z-value = −2.71, *p* = 0.0068 z-value = −2.40, *p* = 0.0163 z-value = −1.81, *p* = 0.0710			

^a^ Means followed by a different lowercase letter within column were significantly different between groups, *p* < 0.05, Tukey’s test.

^b^ Means followed by a different uppercase letter within row were significantly different between moth types, *p* < 0.05, paired *t*-test.

## Data Availability

The raw data supporting the conclusions of this article will be made available by the authors on request.

## References

[1] Knight A.L., Preti M., Basoalto E. (2025). What can we learn from dissecting tortricid females about the efficacy of mating disruption programs?. Insects.

[2] Knight A.L., Preti M., Basoalto E., Mujica M.V., Favaro R., Angeli S. (2021). Combining female removal with mating disruption for management of *Cydia pomonella* in apple. Entomol. Gen..

[3] Knight A.L., Preti M., Basoalto E. (2026). Active assessment of female codling moth, *Cydia pomonella* (L.), mating status under mating disruption technologies. Insects.

[4] Knight A.L., Preti M., Basoalto E. (2025). Factors impacting the use of an allelochemical lure in pome fruit for *Cydia pomonella* (L.) monitoring. Insects.

[5] Wearing C.H. (2022). Life table simulations of a univoltine codling moth, *Cydia pomonella*, population 3. Impact of immigration on the effectiveness of mating disruption. Biocontrol Sci. Technol..

[6] Howell J.F., Clift A.E. (1974). The dispersal of sterilized codling moths released in the Wenas Valley, Washington. Environ. Entomol..

[7] Mani E., Wildbolz T. (1977). The dispersal of male codling moths (*Laspeyresia pomonella* L.) in the Upper Rhine Valley. J. Appl. Entomol..

[8] Schumacher P., Weyeneth A., Weber D.C., Dorn S. (2008). Long flights in *Cydia pomonella* L. (Lepidoptera: Tortricidae) measured by a flight mill: Influence of sex, mated status and age. Physiol. Entomol..

[9] Knight A.L., Preti M., Basoalto E., Rebolledo Ranz R.E. (2025). Sprayable sex pheromones for codling moth: Waiting for grower adoption. Advances in Entomology.

[10] Agrian Label. https://www.agrian.com/labelcenter/results.cfm.

[11] R Core Team (2025). R: A Language and Environment for Statistical Computing.

[12] Kovanci O.B. (2015). Co-application of microencapsulated pear ester and codlemone for mating disruption of *Cydia pomonella*. J. Pest. Sci..

[13] Light D.M., Grant J.A., Haff R.P., Knight A.L. (2017). Addition of pear ester with sex pheromone enhances disruption of mating by female codling moth (Lepidoptera: Tortricidae) in walnut orchards treated with meso dispensers. Environ. Entomol..

[14] Arthurs S.P., Hilton R., Knight A.L., Lacey L.A. (2007). Evaluation of the pear ester kairomone as a formulation additive for the granulovirus of codling moth (Lepidoptera: Tortricidae) in pome fruit. J. Econ. Entomol..

[15] Schmidt S., Tomasi C., Pasqualini E., Ioriatti C. (2008). The biological efficacy of pear ester on the activity of granulosis virus for codling moth. J. Pest. Sci..

[16] Light D.M., Knight A.L. (2011). Microencapsulated pear ester enhances insecticide efficacy in walnuts for codling moth (Lepidoptera: Tortricidae) and navel orangeworm (Lepidoptera: Pyralidae). J. Econ. Entomol..

[17] Knight A.L., Light D.M. (2013). Adding microencapsulated pear ester to insecticides for control of *Cydia pomonella* (Lepidoptera: Tortricidae) in apple. Pest Man. Sci..

[18] Knight A.L., Flexner L. (2007). Disruption of mating in codling moth (Lepidoptera: Tortricidae) by chlorantraniliprole, an anthranilic diamide insecticide. Pest Man. Sci..

[19] Thomson D., Brunner J., Gut L., Judd G., Knight A. (2001). Ten years implementing codling moth mating disruption in the orchards of Washington and British Columbia: Starting right and managing for success!. IOBC WPRS Bull..

[20] Jones V.P., Baker C.C., Wilburn T.D. Movement of codling moth between abandoned and commercial orchards. Proceedings of the Orchard Pest & Disease Management Conference 81.

[21] Sharon R., Tomer M., Avraham A., Harari A.R. (2024). Female codling moths evade the mating disruption control tactic. Res. Sq..

[22] Knight A.L., Koul O., Cuperus G., Elliot N. (2008). Codling moth area wide integrated management. Areawide Pest Management: Theory and Implementation.

[23] McGhee P., Epstein D., Gut L. (2011). Quantifying the benefits of areawide pheromone mating disruption programs that target codling moth (Lepidoptera: Tortricidae). Am. Entomol..

[24] Ioriatti C., Lucchi A. (2016). Semiochemical strategies for tortricid moth control in apple orchards and vineyards in Italy. J. Chem. Ecol..

[25] Witzgall P., Stelinski L., Gut L., Thomson D. (2008). Codling moth management and chemical ecology. Annu. Rev. Entomol..

[26] Knight A.L. (2006). Assessing the mating status of female codling moth (Lepidoptera: Tortricidae) in orchards treated with sex pheromone using traps baited with ethyl (*E,Z*)-2,4-decadienoate. Environ. Entomol..

[27] Tyson R., Newton K.D., Thistlewood H., Judd G. (2008). Mating rates between sterile and wild codling moths (*Cydia pomonella*) in springtime: A simulation study. J. Theor. Biol..

[28] Judd G.J.R., Thistlewood H.M.A., Gardiner M.G.T., Lannard B.L. (2006). Is lack of mating competitiveness in spring linked to mating asynchrony between wild and mass-reared codling moths from an operational sterile insect programme?. Entomol. Exp. Appl..

[29] Judd G.J.R., Arthur S., Deglow K., Gardiner M.G.T. (2011). Operational mark-release-recapture field tests comparing competitiveness of wild and differentially mass-reared codling moths from the Okanagan-Kootenay sterile insect program. Can. Entomol..

[30] Evenden M.L., McClaughlin J.R. (2005). Male oriental fruit moth response to a combined pheromone-based attracticide formulation targeting both oriental fruit moth and codling moth (Lepidoptera: Tortricidae). J. Econ. Entomol..

[31] Il’ichev A.L., Williams D.G., Gut L.J. (2007). Dual pheromone dispenser for combined control of codling moth *Cydia pomonella* L. and oriental fruit moth *Grapholita molesta* (Busck) (Lep. Tortricidae) in pears. J. Appl. Entomol..

[32] Joshi N., Hull L.A., Krawczyk G., Rajotte E.G. (2008). Field results of mating disruption technologies for the control of codling moth, *Cydia pomonella* (L.), and oriental fruit moth, *Grapholita molesta* (Busck) in Pennsylvania apple orchards. Asp. Appl. Biol..

[33] Stelinski L.L., Gut L.J., Haas M., McGhee P., Epstein D. (2007). Evaluation of aerosol devices for simultaneous disruption of sex pheromone communication in *Cydia pomonella* and *Grapholita molesta* (Lepidoptera: Tortricidae). J. Pest. Sci..

[34] Stelinski L.L., Il’ichev A.L., Gut L. (2009). Efficacy and release rate of reservoir pheromone dispensers for simultaneous mating disruption of codling moth and oriental fruit moth (Lepidoptera: Tortricidae). J. Econ. Entomol..

[35] Knight A., Cichon L., Lago J., Fuentes-Contreras E.F., Barros-Parada W., Hull L., Krawczyk G., Zoller B., Hansen R., Hilton R. (2014). Monitoring oriental fruit moth ad codling moth (Lepidoptera: Tortricidae) with combinations of pheromone and kairomones. J. Appl. Entomol..

[36] Knight A.L., Mujica V., Basoalto E., Preti M. (2024). Simultaneous effective monitoring of *Grapholita molesta* and *Cydia pomonella* (Lepidoptera; Tortricidae) in traps with a dual sex pheromone/kairomone lure plus a UV-A light. J. Appl. Entomol..

[37] Knight A.L., Preti M., Basoalto E., Fuentes-Contreras E.F. (2023). Increasing catches of adult moth pests (Lepidoptera: Tortricidae) in pome fruit with low-intensity LED lights added to sex pheromone/kairomone lure-baited traps. J. Appl. Entomol..

